# I could take the judgment if you could just provide the service: non-prescription syringe purchase experience at Arizona pharmacies, 2018

**DOI:** 10.1186/s12954-019-0327-1

**Published:** 2019-09-18

**Authors:** Beth E. Meyerson, Carrie A. Lawrence, Summer Dawn Cope, Steven Levin, Christopher Thomas, Lori Ann Eldridge, Haley B. Coles, Nina Vadiei, Amy Kennedy

**Affiliations:** 1 Southwest Institute for Research on Women, University of Arizona, 925 N. Tyndall Ave, Tucson, AZ 85719 USA; 2 Rural Center for AIDS/STD Prevention, Indiana University School of Public Health-Bloomington, Bloomington, IN 47405 USA; 3 Institute for Research on Addictive Behaviors, Indiana University School of Public Health-Bloomington, 1025 E. 7th Street, Bloomington, IN 47405 USA; 4 Harm Reduction and Foster Care Advocate, Tucson, AZ USA; 5 Sonoran Prevention Works, 3201 N 16th St suite 9, Phoenix, AZ 85016 USA; 6 College of Pharmacy, University of Arizona, 1295 N. Martin Avenue, Tucson, AZ USA

**Keywords:** Syringe access, Hepatitis C, HIV, Stigma

## Abstract

**Background:**

Community pharmacies are important for health access by rural populations and those who do not have optimum access to the health system, because they provide myriad health services and are found in most communities. This includes the sale of non-prescription syringes, a practice that is legal in the USA in all but two states. However, people who inject drugs (PWID) face significant barriers accessing sterile syringes, particularly in states without laws allowing syringe services programming. To our knowledge, no recent studies of pharmacy-based syringe purchase experience have been conducted in communities that are both rural and urban, and none in the Southwestern US. This study seeks to understand the experience of retail pharmacy syringe purchase in Arizona by PWID.

**Methods:**

An interview study was conducted between August and December 2018 with 37 people living in 3 rural and 2 urban Arizona counties who identified as current or former users of injection drugs. Coding was both a priori and emergent, focusing on syringe access through pharmacies, pharmacy experiences generally, experiences of stigma, and recommendations for harm reduction services delivered by pharmacies.

**Results:**

All participants reported being refused syringe purchase at pharmacies. Six themes emerged about syringe purchase: (1) experience of stigma and judgment by pharmacy staff, (2) feelings of internalized stigma, (3) inconsistent sales outcomes at the same pharmacy or pharmacy chain, (4) pharmacies as last resort for syringes, (5) fear of arrest for syringe possession, and (6) health risks resulting from syringe refusal.

**Conclusions:**

Non-prescription syringe sales in community pharmacies are a missed opportunity to improve the health of PWID by reducing syringe sharing and reuse. Yet, current pharmacy syringe sales refusal and stigmatization by staff suggest that pharmacy-level interventions will be necessary to impact pharmacy practice. Lack of access to sterile syringes reinforces health risk behaviors among PWID. Retail syringe sales at pharmacies remain an important, yet barrier-laden, element of a comprehensive public health response to reduce HIV and hepatitis C among PWID. Future studies should test multilevel evidence-based interventions to decrease staff discrimination and stigma and increase syringe sales.

## Introduction

Community pharmacies are important public health partners because they provide services for a range of health issues [1–3]. Community pharmacies include chain pharmacies (such as CVS), independent pharmacies, food store pharmacies (such as Kroger), or mass merchandisers (such as Walmart). These pharmacies are especially important for rural populations and those who do not have access to the health system because they are found in almost any community [4–7]. Community pharmacies also contribute to the prevention of viral hepatitis and HIV because their services can include hepatitis A (HAV) and hepatitis B (HBV) vaccination [8], sterile syringe dispensing [9, 10], consultation about PrEP (pre-exposure prophylaxis for HIV prevention) [11, 12], the sale of HIV tests, and (in some cases) provision of HIV testing and consultation [13, 14].

Retail sale of syringes through pharmacies is widely recognized as a public good, as only two states (TN and DE) expressly prevent it [15]. That all but two states allow retail syringe sales is a testament to the importance of sterile syringe access to prevent HIV, hepatitis C (HCV), HBV, and other health conditions caused by syringe reuse and/or sharing. However, state policies are not uniform among or even sometimes within states, and some states allow significant latitude for pharmacist discretion [16], while others require cumbersome documentation of personal information [17, 18]. Further, paraphernalia possession laws often conflict with retail syringe laws, implying that prescribed substances are the only allowable purpose for syringe purchase [19].

People who inject drugs (PWID), a subset of those who seek to purchase syringes in retail settings, face significant barriers accessing sterile syringes [20–22]. The impact is shown in health outcomes: HIV seroprevalence among PWID in the USA is 7% [23], and this represents 10% of all HIV infections. For HCV, 58% (*r* 38–68%) of PWID are estimated to be living with HCV [24]. HCV positivity depends upon how long a person has been injecting: between 75 and 90% of people who have injected a long time are HCV-positive, and between 18 and 38% of people who have injected less than 3 years are HCV-positive [25–28]. HCV infections have been increasing more frequently in non-urban areas [29, 30] as evidenced by a surge in new HCV cases from 2011 to 2016 [31].

Despite the significant health need for sterile syringes, the implementation of syringe services programs (SSP) in the USA has been variable. Not every community has an SSP, and even those that operate have limited hours and locations [32, 33]. This is particularly an issue for people in rural areas with limited transportation options [19]. The lack of access may explain why at least 25% of PWID report sharing syringes, and only 52% received their sterile syringes from syringe services programs [22].

The public health importance of and opportunity for syringe access through pharmacy purchase sharpens in view of significant health need, varied implementation of syringe services programs, and the existing law allowing pharmacy syringe sales. It has been argued that the combination of pharmacy syringe sales and SSPs can help to reduce HIV and HCV among PWID [10, 34, 35].

Assuring retail pharmacy syringe access is complicated by pharmacy-level policies, practices, and pharmacy staff behaviors. Taussig et al.’s 2002 study among 20 Atlanta pharmacists found that pharmacist attitudes and beliefs about drug use and policy served as barriers to syringe dispensing [16]. Lutnik et al.’s 2012 study among 11 PWID in San Francisco found that most reported experiencing pharmacy staff judgment because of their drug use and were treated with disrespect when asking to buy syringes [36]. That said, Riley and colleagues in 2010 found that 39% of San Francisco PWID study participants reported purchasing syringes through pharmacies [10]. That same year, Pollini et al. found that 81% of PWID in Tijuana, Mexico, purchased a syringe in the past 6 months, even though 16% were refused or overcharged [37]. Despite these challenges, retail pharmacy syringe sales is an essential and lifesaving component to comprehensive efforts to reduce HIV, HCV, and HBV among PWID. This was observed by Pouget et al.’s 2005 study finding that pharmacy sale of syringes in Harlem and the Bronx was associated with a decrease in receptive syringe sharing among PWID [33].

Experiences with retail syringe purchase are known from studies in California, New York, Colorado, Connecticut, Missouri, Kentucky, and Tijuana; yet, many were conducted over a decade ago. To our knowledge, no studies have been conducted recently (in the last 5 years) and in communities that are both rural and urban. To our knowledge, none have been conducted in the Southwestern US.

This study seeks to understand the experience of retail syringe purchase in Arizona pharmacies by PWID. Arizona is a good location for such a study because it experienced a 40% increase in HCV from 2013 to 2017 [38, 39]. Two of Arizona’s counties were identified nationally as targets for public health policy concern: Mohave county was designated as being among the top 218 US counties vulnerable to an HCV or HIV outbreak [40], and Maricopa County (Phoenix) was prioritized by the 2019 National HIV Plan due to higher rates of HIV transmission [41]. There is no statewide syringe access law in Arizona, though one county, Pima (Tucson) [42], allowed the health department to establish an SSP in 1996. In Arizona, syringes are considered drug paraphernalia if they are intended for the parenteral use of illegal substances as defined under the law [43]. Finally, from a public health investment standpoint, Arizona does not invest strongly in public health. The state per capita public health investment is $9.00 [44]. This places Arizona third from the bottom among US states.

## Methods

An interview study was conducted between August and December 2018. A team of five community-experienced and academic interviewers conducted face-to-face interviews lasting up to 1 hour with people who were 18 years or older, living in Arizona and identifying as a current or former user of injection drugs. Two academic interviewers were PhD-trained harm reduction researchers at Indiana University with over 2 decades of qualitative research experience. Three community-experienced interviewers were former drug users who were trained by the principal investigator (Meyerson) to conduct interviews. All interviewers completed the Social and Behavioral Responsible Conduct of Research course through the Collaborative Institutional Training Initiative and filed conflict of interest disclosure documents through the Indiana University.

Study recruitment was accomplished through word-of-mouth advertisement by harm reduction organizations throughout Arizona, HIV programs, operating syringe service programs (including underground programs), through social networks of people who inject drugs, and by snowball sampling among interview participants.

Interview participants were offered a gift card worth $20.00 for participation. Anonymity in interviews was encouraged for participant protection, and interviews were conducted in a private room. Interviews were audio recorded, transcribed, deidentified as necessary, and checked for accuracy by the principal investigator (Meyerson). Coding was both a priori and emergent, with a focus on syringe access through pharmacies, pharmacy experiences generally, experiences of stigma, and recommendations for harm reduction services delivered by pharmacies. A second researcher (Eldridge) independently coded a sample of interviews for an examination of inter-rater reliability. An initial coding conference was held to identify and manage discrepancies. Two minor coding discrepancies were identified. A final coding scheme emerged and was used for all interviews. Once coding and analysis was completed, a conference was held with the entire study team to confirm observations and to determine priority findings and dissemination of those findings. The study was deemed exempt by the Indiana University Institutional Review Board.

## Results

Thirty-seven [37] people participated in the study. The sample included 18 cis male, 18 cis female, and 1 trans male participant. The median age of the sample was 37 years (*r* 22–69). Participants were mostly white (72%). Fourteen percent (14%) were Native American (full or part), and 14% were multi-racial. Hispanic ethnicity was reported by 19% of participants. Participants were from both urban and rural areas. Urban areas included Tucson in Pima County (29.7%) and Phoenix in Maricopa County (27%). Rural areas included Kingman in Mohave County (24.3%), Sierra Vista in Cochise County (10.8%), and Prescott in Yavapai County (8.1%). A majority of the sample (78%) was currently injecting. Participants reported injecting for a median of 5 years (*r* 1–54, IQR 3–11), and 27% reported living with HCV. The HCV prevalence in this sample is likely conservative, as we did not specifically ask for health information. This information was volunteered in the course of interview conversation.

### Syringe purchase experience

All participants reported experiences purchasing or attempting to purchase syringes at an Arizona pharmacy at some point in the last 2 years. Despite having experience buying syringes or attempting to do so, all reported being refused at least once. Participants indicated that because of syringe sales refusal, pharmacies were not their primary source of sterile syringes. The vast majority of participants (81%) reported being part of a secondary syringe access network where they received and/or provided sterile syringes to others when possible.

Table 1 reports the major themes and exemplar interview statements from participants when asked to describe their experiences buying or trying to buy syringes at Arizona pharmacies. These themes included experiences of stigma and judgment from pharmacy staff, feelings of internalized stigma, inconsistent sales outcomes at the same pharmacy or pharmacy chain, pharmacies as last resort for sterile syringes, fear of arrest for syringe possession, and health risks resulting from syringe refusal.

**Table 1:** Reported experiences buying or attempting to buy syringes at Arizona pharmacies, 2018 (*N* = 37)

Major theme (alpha order)	Exemplar statement (interview #, location)
Experience of stigma and judgment from pharmacy staff	*If you are going in there trying to get syringes, be prepared to not be treated great. ‘Cause all of a sudden they are gonna look at you like, “Oh, that’s what you are here for?” You do not know me, I could very well be diabetic, I suppose. They instantly change their whole demeanor. Not very comfortable at all.* (#4, Kingman)
Feelings of internalized stigma	*It would be like someone like me, heavily tattooed, transgender, walking into a Catholic Church on Sunday. You know what I mean? … That’s the only way I can really describe it, yeah. And with a pentagram shirt on … ..So, that’s how I feel, to walk into any pharmacy.* (#37, Sierra Vista)
Inconsistent sales outcomes at the same pharmacy or pharmacy chain	*There’s been some places where I’ve gone to the pharmacy to get needles and was able to do it without any problem, without them looking down on you or questioning why you are getting it, stuff like that. Then, there’s other times when you (return to the pharmacy and) can just tell that they are automatically assuming what you are going to use it for and you can see that they are prejudiced behind it … .. (They do not always sell to you); it’s kind of 50/50.* (#26, Tucson)
Pharmacies as last resort for sterile syringes	*It’s ... some of them are alright, some of them they look at you like, you know, you are a junkie, we ain’t gonna sell you these syringes because you are probably gonna shoot drugs up with it, so we are not gonna sell you them because apparently we are the only pharmacy around here that carries syringes, or we are the only place you are ever gonna get a syringe from. But, what they do not know is that you usually have a syringe you have used probably 20 times in your pocket, it’s all barbed up, and if you do not get a needle from them, you are gonna just use that one.…. or share a needle.* (#31, Phoenix)
Fear of arrest for syringe possession	*Well I know that it’s illegal to distribute a syringe to someone knowing that they are going to use drugs with it. Like, the person who involved me with syringe access told me it’s a class VI felony, which I think is the lowest degree of felony. I think Arizona has “your body is a container” laws, too. And you could probably be charged with paraphernalia or possession or whatever.* (#10, Prescott)
Health risks resulting from syringe refusal	*Oh, they would just turn me away and ask for some kind of documentation for diabetes or a prescription or something. And then that’s when most people will be afraid to go to try to get some and then they just go use whatever*. (#11, Phoenix)

### Stigma: experienced and internalized

Just treat everybody like you treat the nice elderly woman who’s picking up her arthritis medication. (#38, Sierra Vista)

The most frequent experience reported in the pharmacy while trying to purchase syringes was of stigma in the form of discrimination or judgment expressed by the pharmacy staff. Participants did not differentiate whether stigma was expressed by pharmacy technicians, pharmacists, or both, though it was clear that the person at the counter receiving the request for the syringes enacted the first behavioral response. Participants felt that the expressed stigmatizing behavior was syringe-related because the behavior occurred as soon as they asked for syringes. Pharmacy staff behavior was described as a demeanor change following syringe request.

(I just wish they) weren’t so cold to you instantly. ‘Cause they’re like, “Hey, how can I help you?” “Oh hey, I’m here to get some syringes.” “Oh okay, hold on one minute.” Then it all gets dark, especially if you had to do it directly with a pharmacist. (#4, Kingman)

Participants felt that staff judgment was not necessarily focused on the syringes, per se, because they noted that others purchased syringes for more socially acceptable uses such as diabetes or for medicating their pets. Instead, participants believed that pharmacy staff judged their drug use.

Well, when I would go and ask for syringes, they would kind of look at me funny, like ‘what are you ...’ Then ask me if I had a diabetes card or whatever, and I'm like ‘no,’ and then they’re like ‘well, then you can’t buy them.’ … . Well, I think that when you’re trying to purchase syringes as opposed to just picking up your cough medicine or whatever, they kind of look at you like they don’t want to have nothing to do with you, basically. They’re just really short with you and there’s a lot of definite prejudice there. (#24, Tucson)

Some participants felt that pharmacy staff beliefs were summaries about them as people, as opposed to biases against the behavior of injection drug use. Participants reported feeling like caricatures and judged by pharmacy staff as a class of people. Perceived nonverbal messages were that they were not trustworthy, “not clean,” likely unhoused, and were not conscious about their health. The irony about this last point was noted by one participant when discussing pharmacy staff stigma in the face of great lengths people go to purchase sterile syringes: *I do not understand that. I mean,* (buying syringes) *is like buying rubbers and practicing safe sex. I do not understand it* (#12, Kingman).

At least I’m trying to be safe about it, rather than just, using whatever. But you guys would be a lot more pissed off if there was a giant HIV epidemic, or something, over (not) selling syringes to people. (#34, Sierra Vista)

Participants felt that the summary viewpoint about them was formed at the time of sales refusal and would be carried through subsequent transactions.

I don’t know if blackballed is the right word. But you are now, if you order some syringes, a drug addict. You’re an IV drug user, no matter what, whether you are or not. But that’s the thought and the way they treat you. I mean, there’s a couple of people (pharmacy staff) that I’ve talked to over years since I’ve been here. They were fine after I talked to them for awhile. They’re like, “You don't act like a drug addict.” Well, how do they act? (#13, Kingman)

### Health impact of syringe refusal

Participants indicated that their pharmacy experience influenced whether they would try again to buy syringes at another time. The choices resulting from syringe sales refusal were not only burdensome, but injurious.

“What they don’t know is that you usually have a syringe you’ve used probably 20 times in your pocket. It’s all barbed up, and if you don’t get a needle from them, you’re gonna just use that one.” (#31, Phoenix)

The burdened placed by syringe refusal meant additional driving or a “seek and find” method of syringe access to protect health. This was expressed by participants who had transportation and means of accessing multiple pharmacies.

I remember one time, I had to go to five different (pharmacy chain), running across town just to get a bag of syringes. I know it’s not because they don’t have those (syringes) in the back. I know it’s because whoever’s in the pharmacy has a thing against drug addicts, and thinks we’re the absolute scum of the earth. (#25, Tucson)

Pharmacy syringe purchase outcomes were inconsistent “*50/50*, even at the same pharmacy.” According to participants, this made it difficult for them to form clear opinions about whether pharmacies were good or bad places to obtain syringes. The experiences of enacted stigma by pharmacy staff and inconsistent syringe sales refusal appeared to reinforce decisions to abjure pharmacies. Many participants felt that staff behavior and pharmacy practice was by design and that perhaps pharmacies did not want to help people prevent HIV or HCV if they happened to inject drugs. Beliefs about probable stigmatized treatment also reinforced behavioral outcomes of deciding not to go to the pharmacy to attempt to purchase syringes at all.

As long as they don’t see my arms or my legs, I’m treated like a normal person. If they see my arms and my legs, because I’ll have like a bump or a bruise or some buildup of scar tissue, or abscess that’s healing, it’s kind of embarrassing. They think you’re gross. They think, “Oh, they’re unclean.” (It feels) awful to go to a pharmacy. (#21, Tucson)

In contrast, a few participants reported positive experiences with syringe purchase at pharmacies and expressed surprise about them during the interviews. The reported anticipatory judgment was present, it did not dissuade attempts to purchase, and was somehow mitigated through a positive experience.

For me, it’s pretty much just walk in, walk up to the counter. I only think I felt nervous once when I was trying to hit a pharmacy at like midnight, which is relatively unusual even for me. Actually that was the easiest one I think I ever had of getting some. … . It seems to always come up in their mind if you are just asking for syringes. Is this a drug addict or not? Of course, it’s going to color or change the way they act a little bit. Once I get the syringes, I don’t care personally. (#20, Tucson)

### Law and pharmacy policy

Participants spoke about policy in two ways: (1) in terms of Arizona’s law about syringe purchase and possession and (2) pharmacy policy about syringe sales—the focus of the policy and how it was expressed. All participants understood that it was legal to purchase syringes over the counter, though some felt that the pharmacy would work closely with law enforcement to “force us out.” This was primarily based on their reported experiences with the enforcement of Arizona paraphernalia possession law which deems syringes contraband if intended for injection drug use.

I know with paraphernalia, they’ll charge you immediately with a misdemeanor. I take that back, if it’s a rig, and it’s dirty, it’s a felony. Okay. Now, it usually gets dropped to a misdemeanor depending on your record etc. If it’s clean, they will, I mean they’re not going to take your word for it that it’s clean just because it looks clean doesn’t mean it’s clean. So then they test it, per say. (#1, Kingman)

The fear of arrest was palpable, as participants reported significant concerns about the safety of obtaining sterile syringes and possessing them after leaving the pharmacy.

I would be afraid of cops lurking around wherever they know that people are getting clean syringes……. if I knew that I was putting myself at risk to be arrested, I would totally avoid it if that was a possibility. (#9, Prescott)

There was also the perception that the paraphernalia law extended to pharmacies, even though in Arizona, retail syringe sale is legal. In this example, the participant thought it was a pharmacy policy not to sell syringes based on state paraphernalia law. This participant identified the outcome of the conflict between current retail syringe sales and paraphernalia laws.

The way that laws are written about possession (and) the way that laws are written about what a pharmacist’s responsibility is once they know that you are in possession... once they have a suspicion that you are not using your prescriptions responsibly. They could lose their license if they show you that compassion, so until we cut them a break, I don’t think the junkies are getting a break. (#18, Tucson)

Many participants encountered different pharmacy policies regarding syringe sales, and they experienced inconsistency in their application—whether within the same pharmacy or within the same pharmacy company (such as chain, food store, or mass merchandising pharmacies). Examples of pharmacy policies reported by participants included requirements for identification, requirements for a prescription, evidence of having a health condition that required injection (substance use notwithstanding), and requirements for purchasing a certain amount.

Oh, they would just turn me away and ask for some kind of documentation for diabetes or a prescription or something. And then that’s when most people will be afraid to go to try to get some and then they just go use whatever. (#11, Phoenix)

One participant spoke of a recent pharmacy policy change to sell only boxes of 100 syringes and no longer bags of 10. The cost increase was a sufficient barrier to pharmacy syringe purchase.

It’s automatic, that unless you look like you need them for insulin or something, they just look at you kind of funny. And you’re just like, “Yeah. We all get the point. I’m buying insulin syringes.” …..They want to stop the problem (of syringe purchasing by PWID) and everything by selling boxes now. (#34, Sierra Vista)

The need for identification was not reported by participants as a barrier to retail purchasing. Instead, the primary policy issue for participants was the requirement of a prescription to purchase them. Arizona law is not entirely clear about the level of discretion granted pharmacists related to non-prescription syringe sales and the issuing of additional requirements. Participants reported experiencing these policies inconsistently at the same pharmacy or across a pharmacy chain with multiple locations. For example, in one town, participants might report being able to purchase syringes at Wal-Mart or Walgreens, while in other towns, they could not do so at these same company pharmacies.

There’s been some places where I’ve gone to the pharmacy to get needles and was able to do it without any problem, without them looking down on you or questioning why you’re getting it, stuff like that. Then, there’s other times when you (return to the pharmacy and) can just tell that they’re automatically assuming what you’re going to use it for and you can see that they’re prejudiced behind it….. (They don’t always sell to you); it’s kind of 50/50. (#26, Tucson)

## Discussion

This study is the first in several years to document the personal experience of people buying or attempting to buy syringes at pharmacies for injection drug use in urban as well as rural areas, and likely the first in the Southwestern US. Unlike the studies in New York and San Francisco, experiences reported here are in a state that has not permitted syringe service programming. Therefore, the importance of our findings is heightened by the fact that pharmacies are the only structural option for sterile syringe access for PWID throughout Arizona. Reich et al.’s 2002 focus group study of urban and rural PWID in Colorado, Connecticut, Kentucky, and Missouri found that barriers to pharmacy-based syringe purchase included requirements to purchase larger amounts (packs of 50 or 100), having to craft stories about why syringes were needed (such as being diabetic), and feeling stigmatized by pharmacists when purchasing syringes [45]. Here, we found one requirement for purchase of certain amount (a box vs. a bag) and variously implemented policies requiring a prescription or identification. According to participants, these policies were never publicly explicit.

The primary finding of experienced enacted stigma by pharmacy staff reflects findings by Pollini et al. Here, in Arizona, participants reported internalized and anticipatory stigma about appearance such as “looking like a druggie,” “looking homeless,” or having “tracks” on their arms. As with Pollini, we found that the impact of sales refusal on health choices by PWID was deleterious. Pollini observed that pharmacy syringe sale refusal and overcharging were related to sharing a syringe that had been used at least five times and number of lifetime abscesses [36]. Likewise, in Arizona, participants reported having no choice than to reuse or share syringes when refused syringe purchase at the pharmacy. While this was not a causal observation, the association was powerful, as it reflects the harmful health effects of community pharmacy syringe sale refusal and inappropriate pharmacy policy such as overcharging or requiring syringes to be purchased by the box. The impact on likely healthcare utilization of experienced stigma at the pharmacy and in other healthcare settings by PWID was also found in a similar-sized California study (*N* = 46) by Paquette et al. in 2018 [46].

Every Arizona study participant reported that pharmacy practices and staff stigmatization yielded increased deadly risk behaviors and associated health outcomes for PWID. Our findings were that, in Arizona, community pharmacy practices and staff stigmatization around syringe sales contributed to an increase in injection risk behaviors which would likely lead to associated deadly health outcomes for PWID. The health impact of stigma against PWID has been found by others [47–50], and it bears highlighting again here, because it is clear that structural stigma against people who inject drugs facilitates deadly health and life circumstances particularly in states like Arizona. Similar to Indiana, Arizona faces a concerning increase in HCV. As Gonsalves et al. demonstrated in 2018 [51], had Indiana appropriately responded to the increase in HCV observed in 2010–2011 by enacting syringe access policy as well as increasing HIV and HCV testing and follow up, the 2015 Indiana HIV outbreak among PWID would have been limited to only 52 persons. Today, there are over 230 persons infected with HIV related to the Indiana HIV outbreak [52]. The lack of policy action on behalf of PWID had deadly outcomes in Indiana. Can we prevent this from happening in Arizona and elsewhere?

The confluence of the opioid pandemic and infectious diseases highlights the need for novel, integrated approaches to address HIV and HCV rates, particularly in communities with a scarcity of public health resources. The surge in HCV and the vulnerability to outbreaks of HCV and HIV will continue in Arizona until solutions are identified that capitalize on our existing laws for the benefit of health: it is legal to sell and purchase syringes in a pharmacy without a prescription in Arizona. However, the conflict of this and existing paraphernalia laws may in fact be a barrier to syringe dispensing. These issues underscore the immediate need for evidence-based interventions to change pharmacy practice and public policy for syringe sale and possession, so that syringe sales can help improve the health of Arizonans.

Reflecting Lutnik et al.’s San Francisco study, Arizona participants valued the opportunity to purchase sterile syringes at pharmacies. There are few pharmacy syringe sale interventions beyond Fuller et al.’s 2001 multilevel intervention in New York [53] and Compton et al.’s 2004 multi-state “secret shopper” study of pharmacy syringe purchase [54]. Fuller’s community, social, and pharmacy-level interventions increased pharmacy use by Black PWID in Harlem. Notably, the study followed a change in the New York law. The challenge in Arizona is that the law *already* allows the retail sale of non-prescription syringes. Pharmacies are just not uniformly implementing it. In the case of Compton’s study, 35% of 1600 purchase attempts in Colorado, Connecticut, Missouri, and Kentucky were refused, and this suggests that pharmacy and policy-level interventions are necessary.

The barriers to systemic retail syringe access are likely grounded in personal and structural stigma against people who inject drugs. Reich et al.’s 2002 focus group study of pharmacists found that most pharmacists were ambivalent about syringe sales, and this ambivalence was grounded in lack of information about the impact of sterile syringe access upon HIV and HCV transmission [55]. That said, stigma persisted in Reich’s cohort, and was reflected in the stories told here in Arizona. Helping pharmacies to bridge an important health access gap for Arizonans will be the next task. Our lives depend on it.

## Conclusions

This study identified that in 2018, stigma persists at community pharmacies when people attempt to purchase non-prescription syringes in Arizona. Findings also suggest that stigmatized interaction and sales refusal contributed to syringe risk behaviors that will cause HCV and HIV among PWID. Retail syringe sales at pharmacies remain an important, yet barrier-laden, element of a comprehensive public health response to reduce HIV and HCV among PWID.

## Data Availability

Data from interviews will not be publicly available due to the risks posed to participants. Serious requests to the corresponding author will be considered and reviewed by the research team.
